# A Caspase-Dependent Pathway Is Involved in Wnt/β-Catenin Signaling Promoted Apoptosis in Bacillus Calmette-Guerin Infected RAW264.7 Macrophages

**DOI:** 10.3390/ijms15035045

**Published:** 2014-03-21

**Authors:** Xiaoling Wu, Guangcun Deng, Xiujing Hao, Yong Li, Jin Zeng, Chunyan Ma, Yulong He, Xiaoming Liu, Yujiong Wang

**Affiliations:** 1 Key Laboratory of Ministry of Education for Conservation and Utilization of Special Biological Resources in the Western China, Yinchuan 750021, Ningxia, China; E-Mails: nx_wuxiaol@163.com (X.W.); nx_dgc@163.com (G.D.); haoxiujing@126.com (X.H.); liyong7732@126.com (Y.L.); zengjinnxu@163.com (J.Z.); machnyan0411@163.com (C.M.); heyulong2003@163.com (Y.H.); 2 College of Life Science, Ningxia University, Yinchuan 750021, Ningxia, China

**Keywords:** Wnt/β-catenin signaling, alveolar macrophages, apoptosis, caspase, mycobacterial infection

## Abstract

Apoptosis of alveolar macrophages following *Mycobacterium tuberculosis* infection have been demonstrated to play a central role in the pathogenesis of tuberculosis. In the present study, we found that Wnt/β-catenin signaling possesses the potential to promote macrophage apoptosis in response to mycobacterial infection. In agreement with other findings, an activation Wnt/β-catenin signaling was observed in murine macrophage RAW264.7 cells upon *Mycobacterium bovis* Bacillus Calmette-Guerin (BCG) infection at a multiple-of-infection of 10, which was accompanied with up-regulation of pro-inflammatory cytokines TNF-α and IL-6 production. However, the BCG-induced TNF-α and IL-6 secretion could be significantly reduced when the cells were exposed to a canonical Wnt signaling ligand, Wnt3a. Importantly, the activation of Wnt/β-catenin signaling was able to further promote apoptosis in BCG-infected RAW264.7 cells in part by a mitochondria-dependent apoptosis pathway. Immunoblotting analysis further demonstrated that Wnt/β-catenin signaling-induced cell apoptosis partly through a caspase-dependent apoptosis mechanism by down-regulation of anti-apoptotic protein Mcl-1, and up-regulation of pro-apoptotic proteins Bax and cleaved-caspase-3, as well as enhancement of caspase-3 activity in BCG-infected RAW264.7 cells. These data may imply an underlying mechanism of alveolar macrophages in response to mycobacterial infection, by which the pathogen induces Wnt/β-catenin signaling activation, which in turn represses mycobacterium-trigged inflammatory responses and promotes mycobacteria-infected cell apoptosis.

## 1. Introduction

The infection of *Mycobacterium tuberculosis* (Mtb) is the cause of tuberculosis (TB), which remains a major public health problem in most parts of the world, particularly in developing countries. Despite intensive efforts aimed to understand the underlying mechanism of the interactions of Mtb and host cells, including the alveolar macrophages, our knowledge of the nature between a protective and a pathological host response of Mtb infection is limited [1]. Upon invasion to a host, the Mtb can either be eliminated by host immune responses, lie dormant for a long term of infection that may lead to a latent tuberculosis infection, or progress to active clinical disease by break-down of the immune defense. In the course of Mtb infection, the balance of pro-inflammatory and anti-inflammatory immune responses at the site of infection is crucial for the consequence of host responses [2]. It has been proven that the cell death (apoptotic cell death and necrotic cell death) of macrophage following Mtb infection plays a central role in the pathogenesis of TB. In this context, Mtb infection induces pro-inflammatory cytokines and chemokines, such as interleukin (IL)-6 and tumor necrosis factor-α (TNF-α), by which the TNF-α is capable of subsequently inducing macrophages to produce other cytokines and chemokines, which in turn modulates macrophage apoptosis and necrosis [3].

Apoptosis has been demonstrated to play a pivotal role in host defense against intracellular pathogens, including mycobacterial infection [4]. In spite of an increasing line of evidence revealing that necrosis is a form of cell death with a strictly programmed and ordered series of events, as that seen in an apoptotic cell death, these two distinct modes of programmed cell death appear to have drastically different outcomes for the course of mycobacterial infection [5]. The apoptotic cell death of Mtb-infected macrophages has been recognized as a host strategy to limit mycobacterial growth by deprive the intracellular niches required for bacterial replication [4,6]; while with necrosis, on the other hand, a necrosis-like form of death has been observed and demonstrated to allow the release of viable mycobacteria for subsequent re-infection [7].

Many signaling pathways, including the Wnt/β-catenin pathway, have been evidenced to be involved in the interaction of macrophage and Mtb [8]. The canonical Wnt pathway is initiated by the binding of the Wnt ligand(s) to a receptor complex consisting of a member of the Frizzled (FZD) family and the low-density lipoprotein-receptor-related protein (LRP), which triggers the destruction of regulatory complex of Adenomatous Polyposis Coli (APC)/Axin/glycogen synthase kinase (GSK)-3β by the activation of cytoplasmic adaptor protein disheveled (Dvl) phosphorylation. The displacement of GSK-3β from the APC/Axin/GSK-3β complex subsequently represses its activity, and inhibits the phosphorylation and ubiquitin-mediated degradation of β-catenin, which accordingly increases the accumulation of cytosol β-catenin and lead it translocate into the nucleus, where it interacts with members of the transcription factor family of T-cell factor/lymphoid enhancer factor-1 (TCF/LEF-1) to activate the transcription of Wnt target genes, such as cyclin D1 and c-Myc [9].

The roles of Wnt signaling in the development of immune system and immune cell fate determination, particularly in the T cells have been well documented. However, its role in the modulation of immune response and cell fate determination of immune cells in response to various pathogen infections remains elusive [10–12]. Neumann *et al.* recently demonstrated that the canonical Wnt ligand, Wnt3a was able to promote anti-inflammatory activities in murine Bone marrow-derived macrophages (BMDMs) in response to Mtb infection, in part through a mechanism of up-regulation of FDZ-1 and down-regulation of Toll-like receptor (TLR)/nuclear factor-κB (NF-κB) pathway, by which the Wnt/β-catenin signaling was able to prevent excessive inflammatory response upon Mtb infection [8]. In alveolar epithelial cells, the canonical Wnt signaling has been linked to the development of pulmonary fibrosis, in which an enhanced Wnt signaling augmented the secretion of IL-1β and IL-6 in alveolar type II cells, and reactivate inflammation [13]. Moreover, such activation of Wnt/β-catenin signaling was also found to enhance cell apoptotic potency in melanoma [3]. These findings may imply that the Wnt signaling plays a modulatory role in the inflammatory responses and cell fate determination of host cells in response to a pathogen invasion. Thus, the objective of this study is to explore whether an activation of Wnt/β-catenin signaling is capable of altering the cell fate of macrophage in response to Mtb infection. To this end, we examined the impacts of Wnt//β-catenin signaling on the apoptosis in murine macrophage RAW264.7 cells following *Mycobacterium bovis* Bacillus Calmette-Guerin (BCG) infection.

## 2. Results and Discussion

### 2.1. Results

#### 2.1.1. Bacillus Calmette-Guerin (BCG) Activated Canonical Wnt Signaling in Macrophage RAW264.7 Cells

**Figure 1.**
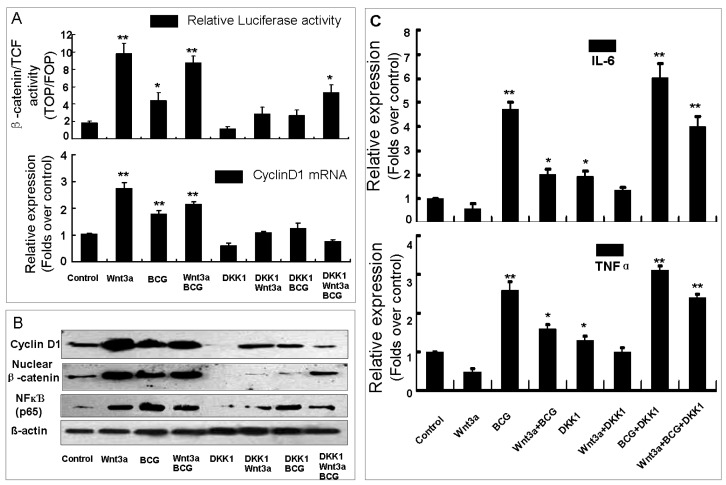
The impacts of Wnt/β-catenin signaling in RAW264.7 cells in response to BCG infection. (**A**) The impact of BCG infection on macrophages was first ascertained by evaluating the relative firefly luciferase activity and the expression of Wnt target gene Cyclin D1 in RAW264.4 cells that transfected with β-catenin/TCF reporter TOPflash and treated with indicated conditions. In the absent of BCG stimulation, RAW264.7 cells showed a weak baseline Wnt signaling activity. However, the Wnt signaling activity could be dramatically elevated by Wnt3a and the BCG infection, and repressed by a Wnt signaling inhibitor, DKK1; (**B**) Immunoblots showed the expressions of Wnt target gene Cyclin D1, nuclear β-catenin and. P65 subunit of NFκB; (**C**) An ELISA analysis of the supernatants of RAW264.7 cells treated with indicated conditions showed the impacts of Wnt3a signaling on the productions of IL-6 and TNF-α in macrophages. Compared to a naïve control, * *p* < 0.05; ** *p* < 0.01. Data represented the mean ± SD from three independent triplicated experiments (*n* = 15).

To evaluate the alteration of Wnt/β-catenin signaling in macrophages in response mycobacterial infection, the murine alveolar RAW264.7 macrophage cells were exposed to Wnt3a-CM or control-CM and co-transfected with β-catenin/TCF response reporter TOPflash or its control FOPflash with DKK1 or pcDNA3.1 plasmids, infected with BCG, or a combination of above treatments (Figure 1). Dual-luciferase assay demonstrated a relative low baseline of Wnt/β-catenin signaling activity in the naïve RAW264.7 cells (data not shown), however, an augmentation of Wnt signaling was detected in the cells exposed to medium contain Wnt signaling ligand, Wnt3a protein (Wnt3a-CM) alone or upon BCG infection (*p* < 0.01) (Figure 1A), which was accompanied by increased levels of nuclear β-catenin and Wnt target gene Cyclin D1 proteins (Figure 1B). In contrast, cells transfected with plasmid expressing Wnt signaling inhibitor DKK1 showed a reduction of Wnt activity in this type of cells (Figure 1A), with decreased abundant nuclear β-catenin and Cyclin D1 expressions in both Wnt3a treated and BCG-infected cells as determined by a immunoblotting assay or qRT-PCR assay (Figure 1A,B). Intriguingly, the BCG-infected cells did not display a synergic or an additive activation of Wnt/β-catenin signaling when they were exposed to Wnt-CM (Figure 1A). This data unambiguously suggested that Wnt/β-catenin signaling was activated in RAW264.7 macrophages upon BCG infection, which was consistent with the previous finding from other group [8].

#### 2.1.2. Wnt/β-Catenin Signaling Down-Regulates BCG-Induced IL-6 and TNF-α Production in RAW264.7 Cells

In order to explore the impact of Wnt/β-catenin signaling on inflammatory responses of macrophages against BCG stimulation, the production of pro-inflammatory cytokines IL-6 and TNF-α was determined by an ELISA in the culture medium of RAW264.7 cells. In line with the findings by others [8], the addition of Wnt3a significantly down-regulated the BCG-induced production of IL-6 and TNF-α in both naïve macrophages and BCG-infected cells (Figure 1C). Contrastly, cells enforced expression of DKK1 exhibited an opposite function of Wnt3a in RAW264.7 cells, where the expression of DKK1 showed an ability to revise the Wnt3a-repressed IL-6 and TNF-α productions in both of uninfected and BCG-infected cells (Figure 1C). Of note, an increased abundance of p65 NFκB subunit protein was found in the naïve RAW264.7 cells exposed to Wnt3a; in contrast the non-infected cells transfected with DKK1 showed a slightly decresed expression of p65 NFκB (Figure 1B). However, the addition of Wnt3a was able to suppress the BCG-induced p65 NFκB expression; and the introduction of DKK1 exhibited a capacity to inhibit Wnt3a induced p65 NFκB expression in the naïve cells, dispite it failed to provoke p65 NFκB expression further in BCG-infected macrophages (Figure 1B). Together with the observations from others, this finding supported the notion of a negative regulatory role of Wnt/β-catenin signaling in host inflammatory responses against mycobacterial infection in macrophages [9].

#### 2.1.3. Wnt3a Promotes Apoptosis in RAW264.7 Cells Infected with BCG

**Figure 2.**
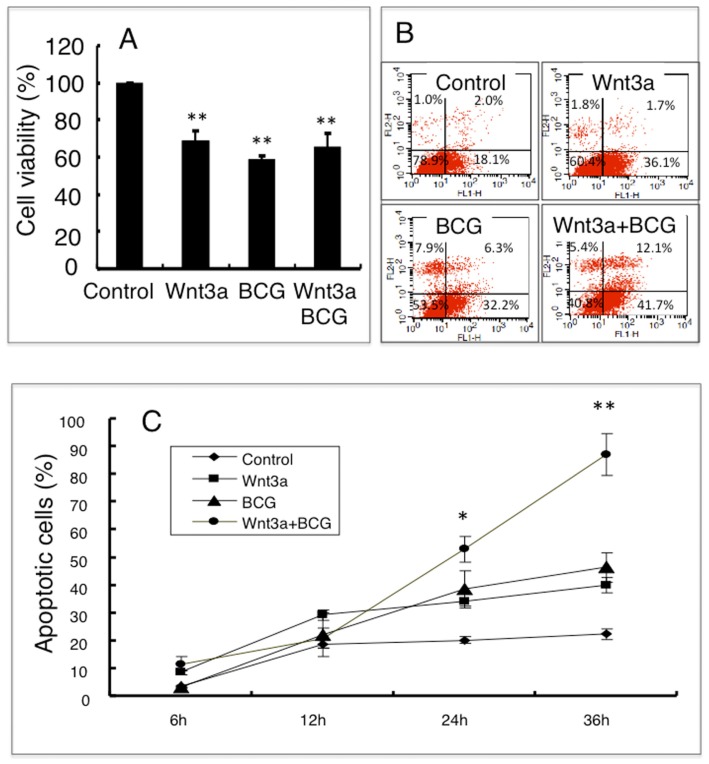
The activation of Wnt/β-catenin signaling promotes BCG-infected cell apoptosis. (**A**) An MTT assay determined the cell viability of RAW264.7 cells treated with indicated conditions for 24 h; (**B**) Representatives of dot plot from five independent experiments of flow cytometry analysis of the apoptotic fraction of RAW264.7 cells treated with indicated conditions for 24 h; (**C**) A time-dependent apoptotic cell death fraction of RAW264.7 cells treated with indicated condition for different time points. Compared to a naïve control, * *p* < 0.05; ** *p* < 0.01. Data represented the mean ± SD from five independent triplicated experiments (*n* = 15 in A; *n* = 9 in B and C).

**Figure 3.**
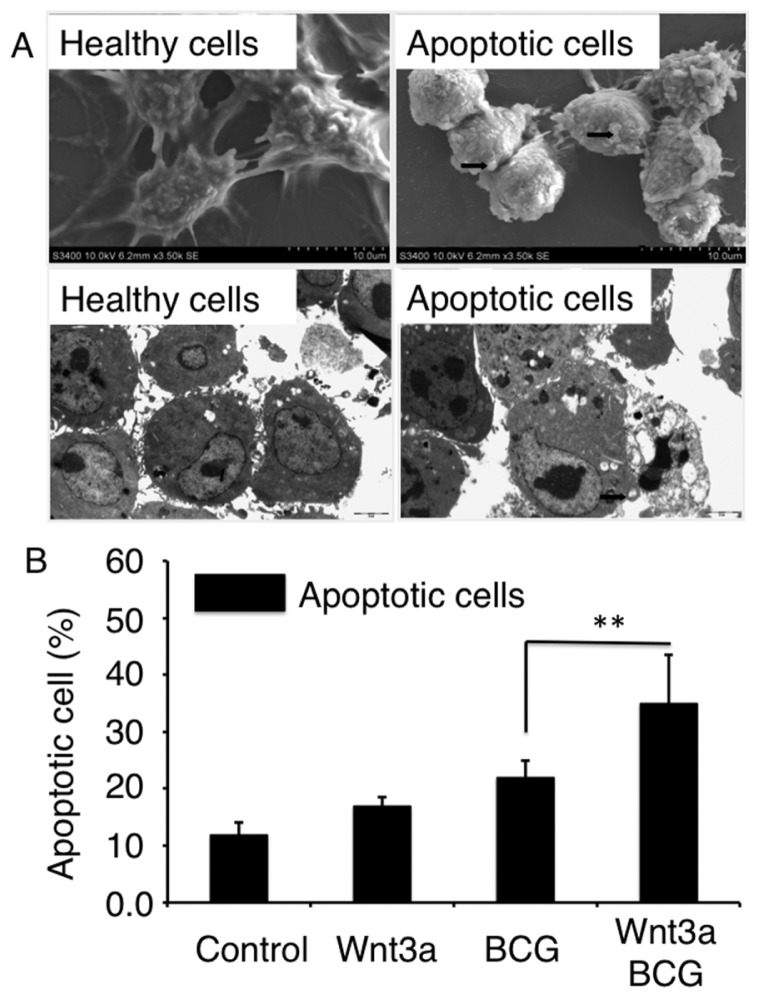
Morphological analysis of the impact of Wnt3a on BCG-infected RAW264.7 cells apoptosis. RAW264.7 cells exposed to Wnt3a-CM or control-CM, followed by infection of BCG at MOI of 10 for 24 h. (**A**) Representative images of SEM (**top panel**) and TEM (**bottom panel**) of healthy RAM264.7 cells (**left panel**) and apoptotic cells (**right panel**); (**B**) Percentages of cells with an apoptotic phenotype as determined by morphology using EM images. Compared to a naïve control, ** *p* < 0.01. Data represented the mean ± SD from three independent triplicated experiments (*n* = 9). Bar in SEM images = 2 μm; Bar in TEM images = 10 μm. Arrows in A indicated apoptotic bodies.

We next sought to investigate whether the canonical Wnt signaling could affect on cell death in macrophages following mycobacterial infection. The MTT assay demonstrated that the viability of cells was significantly reduced by the treatments of Wnt3a-CM and/or BCG infection (Figure 2A). Flow cytometry analysis further revealed an increased frequency of apoptosis cells in the BCG-infected RAW264.7 cells that exposed to Wnt3a-CM, in comparison with the cells treated with other conditions (Figure 2B,C); such a Wnt3a-mediated apoptosis was time-dependent, which was significantly increased at the time after 24 h relative to the control-CM treated and/or uninfected cells (*p* < 0.01) (Figure 2C). The apoptotic characteristics was further confirmed by morphological analysis using electronic microscopy (EM) (Figure 3A), EM images of RAW264.7 cells revealed that the majority of cells exposed to control-CM showed healthy morphology including an integrity of nuclear membrane with abundant surrounding microvilli, uniform cytoplasm with rare cytoplasmic vacuoles, well-organized organelles, nuclei with clear membrane bounder, and uniform speckled distributed chromatin (Figure 3A and data not shown); while increasing numbers of apoptotic cells was observed in cells exposed to Wnt3a-CM or BCG infected, which were characterized with loss of microvilli, mitochondrial swelling, protrusion of plasma membrane (blebs) with apoptotic bodies, as well as nuclear condensation (Figure 3A,B and data not shown); importantly, the addition of Wnt3a-CM significantly promote the BCG-infected cells to an apoptotic cell death, in comparison with the control-CM treated cells when the apoptotic cell numbers were determined by EM morphology (*p* < 0.01) (Figure 3B). These morphological results provide further evidence that activation of Wnt/β-catenin may promote apoptosis in mycobacteria-infected macrophages.

#### 2.1.4. Wnt3a Affects Mitochondrial Membrane Potential (ΔΨm) in RAW264.7 Cells

**Figure 4.**
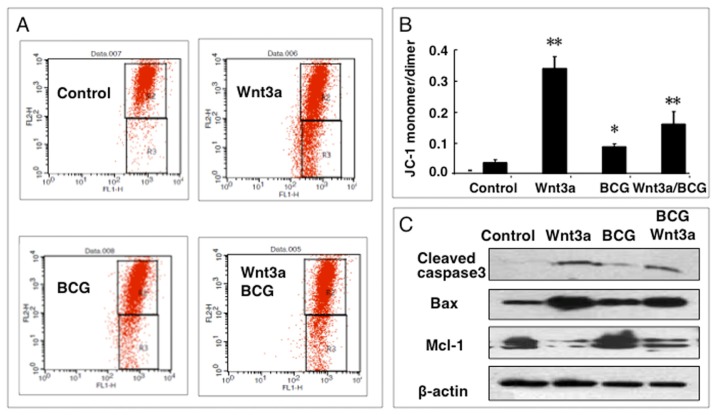
Impact of Wnt/β-catenin signaling on mitochondrial membrane potential (ΔΨm) of RAW264.7 cells. (**A**) Representatives of dot plot of flow cytometry analysis for ΔΨm of cells treated with indicated condition for 12 h; (**B**) fractions of cells with low ΔΨm treated with indicated conditions for 12 h; (**C**) Immunoblots of apoptosis-related proteins in RAW264.7 cells treated with indicated conditions for 24 h. Compared to a naïve control, * *p* < 0.05; ** *p* < 0.01. Data represented the mean ± SD from three independent duplicate experiments (*n* = 9).

The mitochondrial damage is an intrinsic pathway best known for apoptosis [14]. We thus examined the impact of Wnt3a on mitochondria of RAW264.7 cells by ascertaining the mitochondrial membrane potential (ΔΨm). The cells were exposed to Wnt3a, BCG, or BCG plus Wnt3a for 12 h, prior to be stained with JC-1 and analyzed for ΔΨm by flow cytometry assay (Figure 4A). Experimental results showed an ability of Wnt3a to significantly reduce ΔΨm in both naïve RAW264.7 cells and the BCG-infected cells (*p* < 0.01), as compared with the control-CM treated cells (Figure 4B). The infection of BCG also caused a statistical reduction of ΔΨm in RAW264.7 cells (*p* < 0.05) (Figure 4B). Molecular analysis by Western blotting assay further revealed that the Wnt3a-induced apoptosis was accompanied with up-regulation of pro-apoptotic proteins Bax and cleaved caspase-3, and down-regulation of anti-apoptotic proteins Mcl-1macrophages (Figure 4C). Of note, the infection of BCG also caused a statistically different decline of ΔΨm in comparison with the naïve control (*p* < 0.05) (Figure 4B), but no alteration of Mcl-1 expression was detected by an immunoblotting assay (Figure 4C). This result implied that the BCG-induced cell death might employ a different process from that of Wnt3a.

#### 2.1.5. Wnt3a Promoted BCG-Infected RAW264.7 Cell Apoptosis in Part through a Caspase-Dependent Mechanism

**Figure 5.**
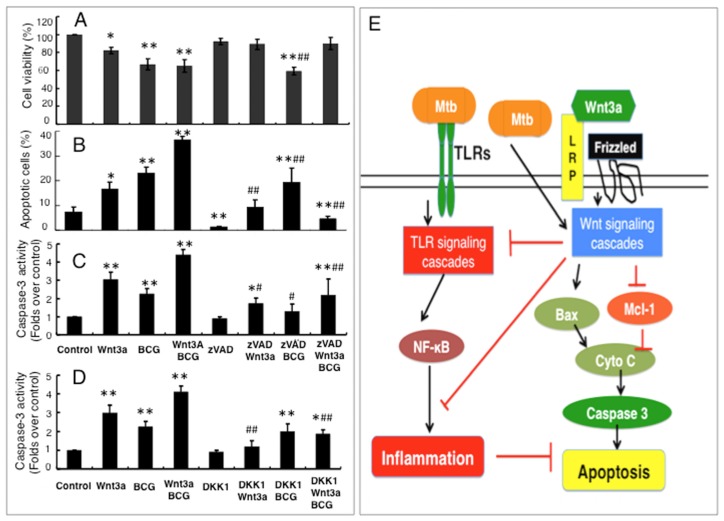
Wnt/β-catenin promotes BCG-infected RAW264.7 cell apoptosis is in part through a caspase-dependent pathway. A caspase-dependent apoptotic pathway was examined in the RAW264.7 cells treated with Wnt3a, BCG, pan caspase inhibitor zVAD, and/or Wnt signal inhibitor DKK1. (**A**) Cell viability determined by an MTT assay for RAW264.7 cells treated with indicated conditions for 6 h; (**B**) Apoptotic cell fraction determined by a flow cytometric assay for RAW264.7 cells treated with indicated conditions for 24 h; (**C**) Relative caspase activity of RAW264.7 cells treated with indicated conditions containing pan caspase inhibitor zVAD for 24 h; (**D**) Relative caspase activity of RAW264.7 cells treated with indicated conditions containing Wnt signal inhibitor DKK1 for 24 h. Compared to a naïve control, * *p* < 0.05; ** *p* < 0.01; compared to the cells treated with zVAD (C) or DKK1 (D) alone, ^#^ *p* < 0.05; ^##^ *p* < 0.01. Data represented the mean ± SD from three independent triplicate experiments (*n* = 15); and (**E**) A proposed mechanism whereby the regulatory role of Wnt/β-catenin signaling in the apoptosis macrophages upon mycobacterial infection. In this model, upon invasion of mycobacteria, the pathogen is first recognized by TLRs of the cells, which in turn triggers the TLR signaling cascade to initiate inflammatory response and activates Wnt/β-catenin signaling that plays a negative feedback inhibitory role in modulation of inflammatory responses. Meanwhile, the activation of the Wnt/β-catenin pathway subsequently promotes apoptotic cell death in the mycobacteria-infected macrophages, in part through a caspase-dependent apoptosis pathway.

To further examine whether the caspase-dependent mechanism was involved in the Wnt3a promoted BCG-infected cell apoptosis, pan caspase inhibitor zVAD was employed. As shown in Figure 5A, the addition of zVAD (20 μm/L) had no impact on cell survival of control group, while increased survival was observed in the cells that exposed to Wnt3a or Wnt3a/BCG, as determined by an MTT assay. Meanwhile, the zVAD could significantly inhibit the Wnt3a- and Wnt3a/BCG-induced apoptotic cell death, (Figure 5B), along with a decreased caspase activity (*p* < 0.01) (Figure 5C), but had no effect on cell death caused by BCG alone (Figure 5B); moreover, the infection of BCG or Wnt3a could partial reverse the zVAD-repressed cell apoptosis with enhanced caspase activity (Figure 5C), implying that Wnt3a and BCG employed a distinct mechanism to induce cell death, and other caspase-independent apoptosis pathway(s) might be also involved in mycobacteria-infected macrophages; however, the addition of Wnt3a further increased the fraction of BCG-induced apoptotic cell death in the cells exposed to zVAD and BCG, relative to the control medium (*p* < 0.01) (Figure 5B). Importantly, such Wnt3a and/or BCG-induced caspase activity could be reduced by the introduction of Wnt signaling inhibitor DKK1 (Figure 5D), suggesting a caspase-dependent apoptosis pathway was involved in this mode of cell death promoted by Wnt/β-catenin signaling. These results clearly indicated that the Wnt3a-promoted BCG-infected RAW264.7 cell apoptosis was in part through a caspase-dependent pathway.

### 2.2. Discussion

Alveolar macrophages are main targets of Mtb infection, which are able to provide critical intracellular niches for Mtb establishing infection in host [4]. It has been demonstrated that the mode of cell death of macrophages following Mtb infection play a central role in the pathogenesis of TB, in which the apoptotic death of the infected cells is likely associated with the elimination of Mtb, while necrotic cell death may facilitate the dissemination and transmission of pathogens [5,7]. Therefore, the balance of these two modes of cell death in macrophages may be an important mechanism that controls the course of Mtb infection [5]. In this study, we demonstrated that the Wnt/β-catenin signaling possessed a potent activity capable of promoting the BCG-induced macrophage apoptosis, at least in part through a caspase-dependent apoptosis pathway.

It is well known that Wnt signaling is capable of governing cell survival, proliferation, differentiation, and apoptosis through multiple intracellular signaling pathways [9]. Several studies have demonstrated that Wnt signaling induced cell proliferation or apoptosis through a pro-inflammation pathway. In an early study on endothelial cells, Masckauchan *et al.* found Wnt/β-catenin signaling promoted cell proliferation by the induction of pro-inflammatory cytokine IL-8 [15]. Such pro-inflammation-mediated and Wnt/β-catenin-promoted proliferation and apoptosis were also been reported in hepatocellular carcinoma (HCC) cells [16,17]. On the other hand, increasing evidence has demonstrated its ability to modulate inflammatory processes in many types of cells, including the dendritic cells (DCs) and macrophages [8,12,18–20]. In terms of mycobacterial infection, an enhanced Wnt signaling was observed in alveolar macrophages [8,18,19]. The expression of a non-canonical Wnt signaling ligand, Wnt5a was found to be increased in macrophages upon mycobacterial infection, which in turn regulated the inflammatory responses via its receptor FDZ5 [18,19]; further study also revealed a TLR signaling-dependent activation of canonical Wnt (Wnt/β-catenin) signaling, along with an augmentation of Wnt3a receptor FZD1 and an inhibition of GSK3β activity in the mycobacteria-infected macrophages [8]. In this regard, the addition of Wnt3a showed an alleviation of inflammatory response with reductions of IL-6 and TNF-α production, an indicative of an anti-inflammatory role of Wnt3a in macrophages [3,8]. In line with these findings, we found that the infection of BCG led an activation of Wnt/β-catenin signaling in RAW264.7 macrophages, which in turn suppressed the activity of TLR signaling-mediated inflammatory responses, subsequently reduces the production of pro-inflammatory cytokines, including the IL-6 and TNF-α. Such notion was also supported by the experiment in RAW264.7 cells exposed to Wnt3a in this study. Intriguingly, Wnt3a was able to provoke the expression of p65 subunit of NFκB in the naïve cells, but suppress its expression in BCG-infected cells, and sequentially repress the productions of cytokines, such as IL-6 and TNF-α, in both of naïve and BCG-infected macrophages in the present study. This finding was consistent with a previous study by Schaale *et al.*, in which they found that Wnt3a was able to negatively regulate inflammatory responses by inhibiting TNF-α production, and the Wnt3a only had a transient effect on the phosphorylation of NFκB p65 subunit [18,19]. Indeed, the TNF-α is an important cytokine capable of inducing macrophages to produce other cytokines and chemokines, and modulating macrophage apoptosis and necrosis [3].

Apart from its immunorepressive role in immune effector cells, the canonical Wnt signal has been demonstrated to possess a property of regulation of cell proliferation and apoptosis in a cell-context dependent manner, by which the activated Wnt/β-catenin signaling was able to enhance cell proliferation but also induce apoptosis in a variety of cells [3,21]. In hematopoietic progenitor cells (HPCs), an activation of the Wnt/β-catenin signaling could trigger a mitochondria dependent apoptotic pathway and promote apoptosis of HPCs by enhancing caspase-3 activity and suppressing anti-apoptotic protein Bcl-2 expression [21], such a Wnt-induced cell apoptosis was also found in mouse intestinal epithelia cells [22] and fibroblast [23]. However, the regulatory role of Wnt/β-catenin signaling in cell fate determination remains controversial. For instance, evoking Wnt/β-catenin signaling by enforced expression of active β-catenin, addition of Wnt3a or inhibition of GSK3β showed an enhanced self-renewal capacity of HPCs *in vitro* and *in vivo*; and an inhibition of Wnt signaling repressed HPC proliferation and reduced their reconstitution capacity [24–26]. Conversely, other studies found that a constitution of β-catenin led to loss the capacity of self-renewal and multiple lineage differentiation in HPC [27,28]. In the present study, we also revealed that Wnt3a could promote BCG-infected RAW264.7 cells apoptosis with its ability to decrease mitochondrial membrane potential in both of naïve cells and the mycobacteria-infected cells, suggesting a capacity of Wnt/β-catenin signaling in the regulation of mitochondria-dependent apoptosis pathway. However, BCG infection alone also caused a reduction of mitochondrial membrane potential, but with no alteration of Mcl-1 expression in RAW267.4 cells, suggested that the BCG-induced cell death might employ a different process from that of Wnt3a. In addition, zVAD failed to reverse BCG-induced cell death, but it could improved cell survival in cells treat with Wnt3a- or Wnt3a/BCG, these results implied that other mechanisms might play major roles in BCG-induced cell death, which need to be further defined in the future.

Caspase-3 is a key effector capable of amplifying apoptotic signals from initiator caspases (such as caspase-8) in an apoptotic pathway [29]. Our data showed that Wnt3a exhibited an ability to promote an apoptotic cell death in BCG-infected RAW264.7 macrophages. The immunoblotting assay further revealed that such increased apoptosis was accompanied with an up-regulation of caspase-3 signaling cascade with an increased expression of pro-apoptotic protein Bax and a decreased expression of anti-apoptotic protein Mcl-1. Such a caspase-dependent Wnt/β-catenin induced apoptosis was further supported by the result from experiments using pan caspase inhibitor zVAD and Wnt inhibitor DKK1, in which the addition of Wnt3a was able to restore the zVAD-repressed caspase-3 and promote BCG-infected cell apoptosis in RAW264.7 cells, and the introduction of DKK1 could inhibit Wnt-activated caspase activity. Of note, BCG infection alone also marginally induced RAW264.7 cell apoptosis, one possible reason for which could be the infection of BCG activated Wnt/β-catenin signaling, which in turn promoted the cell apoptotic death. These data strongly suggest that the Wnt/β-catenin signaling promoted-apoptosis in mycobacteria-infected alveolar macrophages was at least in part through a caspase-dependent apoptosis pathway. Such a notion was also reported in melanoma cell lines, in which Wnt/β-catenin signaling provoked pro-apoptotic protein expression and diminished expression of anti-apoptotic proteins, and inhibition of Wnt signaling by β-catenin siRNAs showed a block of cell apoptosis [3]. Such caspase-dependent Wnt/β-catenin-induced cell apoptosis was also reported in a study using hepatocellular carcinoma (HCC) cells, in which Li *et al.* found that IFNγ could promote HCC cell apoptosis more rapidly in the cells with high levels of β-catenin, as compared with cells with low β-catenin levels, along with markedly activated caspases 3, 8, and 9 [17]. In addition to the caspase-dependent pathway, Wnt/β-catenin signaling was also reported to stimulate cell proliferation via up-regulation of Wnt target gene c-Myc expression in adult human Sertoli cells [30], and in murine teratocarcinoma P19 cells [31]; moreover, the canonical Wnt signaling was capable of promoting cell survival and proliferation through pathways including the AKT/PKB [32], PTEN/PI3K/Akt [33], and MAPK signaling pathways [34].

## 3. Experimental Section

### 3.1. Cell Lines and Wnt3a Conditioned Medium

Murine macrophage RAW264.7 cell line was purchased from shanghai Institute of Biochemistry and Cell Biology, Shanghai, China); the Wnt3a producing cell line, L Wnt3a (overexpressing mouse Wnt3a, ATCC #CRL-2647) and its control L cell line (ATCC #ATCC #CRL-2648) were purchased from American Type Culture Collection (ATCC) (Masassas, VA, USA). The cells were cultured and maintained at 37 °C in a humidified atmosphere of 5% CO_2_ and 95% air in DMEM medium (Invitrogen, Carlsbad, CA, USA) supplemented with 10% Fetal Bovine Serum (FBS) and 1% pen/strep. The L Wnt3a and control L cells were grown to confluency prior to be refreshed with DMEM/2% FBS and kept for 12 h. The culture media were collected and used for preparation of Wnt3a-conditioned medium (Wnt3a-CM) and control medium (control-CM), respectively.

### 3.2. Plasmids and Transfection

The canonical Wnt reporter plasmid carrying 7 TCF binding sites upstream of a minimal c-*fos* promoter driving the firefly luciferase gene (TOPflash) and its control plasmid (FOPflash) were from Millipore (Millipore, Billerica, MA, USA). The transfection control plasmid expressing renilla luciferase was a product of Promega (Promega, Madison, WI, USA). The plasmid expressing Wnt inhibitor DKK1 pCS2-hDKK1-flag (Cat. #15494) was purchased from Addgene (http://www.addgene.com, Addgene, Cambridge, MA, USA). For DNA transfection, RAW264.7 cells were seeded in 6 or 48 well plates and cultured for 18–24 h, and 80%–90% confluent cells were used for transfection. The transfection was performed using FuGENE^®^ HD transfection reagent per manufacturer’s instruction (Roche Applied Science, Basel, Switzerland). The caspase inhibitor carbobenzoxy-valyl-alanyl-aspartyl-[*O*-methyl]-fluoromethylketone (Z-VAD-FMK, zVAD) (Sigma, St. Louis, MO, USA) was dissolved in Dimethyl sulfoxide (DMSO) at a concentration of 20.0 mmol/L as stock solution.

### 3.3. Infection of RAW264.7 Macrophage Cells with BCG

*Mycobacterium bovis* BCG, Beijing strain (Center for Disease Control and Prevention (CCDC), Beijing, China) was grown at 37 °C with shaking in Middle-brook 7H9 broth (BD Diagnostic Systems, Sparks, MD, USA) containing 10% albumin dextrose catalase supplement (Difco, West Molesey, Surrey, UK) for 2 weeks. Cultures were then harvested by centrifugation at 500× *g* for 10 min and re-suspended in the medium. Aliquot of the stock were stored at −80 °C freezer. The above control or transfected RAW264.7 cells were infected with BCG at a multiplicity of infection (MOI) of 10 and incubated at 37 °C in a 5% CO_2_, humidified air atmosphere for additional 6 h prior to being harvested for analysis.

### 3.4. Dual-Luciferase Reporter Assay

The Wnt/β-catenin activity was accessed by a dual luciferase reporter assay using a Dual-Glo (TM) Luciferase Assay System (Promega, Madison, WI, USA) on a 20/20n Luminometer (Turner BioSystems, Sunnyvale, CA, USA) essentially according to the manufacturer’s protocols.

### 3.5. Quantitative Reverse Transcription PCR (qRT-PCR)

Total RNA from cultured cells was isolated using the RNA purification kit per manufacturer’s instruction (TransGen Biotech, Beijing, China). High quality RNA was used for reverse transcription of the first-strand cDNA synthesis by reverse transcription using M-MLV reverse transcriptase (TransGen Biotech, Beijing, China). The quantitative real-time RT-PCR (qRT-PCR) was performed in the BIO Rad IQ5 using SYBR Green I kit (Promega, Madison, WI, USA); the thermal cycling condition for PCR was 95 °C for 30, 40 cycles of 95 °C for 5 s, 61 °C for 30 s, and 72 °C for 30 s, followed by 40 cycles of 55 °C for 15 s. The primers used for qRT-PCR were as follows: β-actin Forward primer (Fw) 5′-TGAGAGGGAAATCGTGCGTGACAT-3′, Reverse primer (Rv) 5′-ACCGCTCGTTGCCAATAGTGATGA-3′; Cyclin D1 Fw 5′-GCAAGCATGCACAGACCTT-3′, Rv 5′-GTTGTGCGGTAGCAGGAGA-3′. The β-actin endogenous control was included to normalize each reaction with respect to RNA integrity, sample loading, and inter-PCR variations. The relative expression ratio was calculated from real-time PCR efficiencies and the crossing point deviation of experimental samples *vs.* controls. The specificity of PCR was determined by sequencing of the PCR products.

### 3.6. Enzyme-Linked Immunosorbent Assay (ELISA)

To determine the concentrations of IL-6 and TNF-α in the medium of macrophage cultures, the RAW264.7 culture medium was collected after treatment and centrifuged at 1000× *g* for 5 min to pellet the cell debris. The supernatant was harvested and stored at −80 °C prior to analysis. The concentrations of murine IL-6 and TNF-α in the supernatant were determined with appropriate ELISA kits per the manufacturer’s instructions (Neobioscience Tech, Shenzheng, China). The protein concentration was determined by comparing the standard protein provided in the kits and expressed as pg/ml. The data was presented as the change of experimental group over the controls.

### 3.7. MTT Assay

Prior to being treated with different conditions, 5 × 10^4^ cells/well were seeded in a 96-well and culture overnight. Cell viability was determined using an MTT (3-(4,5-dimethyl-thiazol-2-yl)-2,5- diphenyl-tetrazolium bromide) assay. Briefly, 100 μL MTT (5 mg/mL in 1 M phosphate-buffered saline, pH 7.6) was added to each well of 96-well plate at the end of each treatment; the plates were then incubated for 2 h at 37 °C in 5% CO_2_ and 95% air. Optical density (OD) was measured at 570 nm with a microplate reader.

### 3.8. Flow Cytometry Analysis for Cell Apoptosis

Cells were treated with different conditions for 2 h prior to be infected with BCG for additional 6, 12, 24, or 36 h, before they were collected and stained with Annexin V and PI using an Apoptosis Detection Kit I (BD Pharmigen, San Jose, CA, USA) for flow cytometry analysis. The flow cytometry analysis was performed on a BD FACSCanto II, and data was analyzed with FlowJo 8.8.6 software (Tree Star Inc., Ashland, OR, USA). All experiments were performed with biological triplicates and data are representative of at least three independent experiments.

### 3.9. Electronic Microscopy

The cells cultured under different conditions were first observed under an inverted microscope before being harvested for electronic microscopy analysis. For scanning electron microscopy (SEM) analysis, the cells were fixed with 2.5% glutaraldehyde, stained with 1.25% osmium tetroxide in PBS, dehydrated, and sputter coated prior to visualization on a Hitachi S-450 microscope (Tokyo, Japan); for transmission electron microscopy (TEM) analysis, the cells were fixed and stained as SEM, followed by infiltration with Spurr resin following dehydration. 80 nm serial sections were then viewed on a Hitachi H-7650 Electron Microscope (Tokyo, Japan). The apoptosis of cells was determined according the morphological criteria described in a previous study [35].

### 3.10. Mitochondrial Membrane Potential (ΔΨm) Assay

The ΔΨm was determined using the dual-emission mitochondrion-specific lipophilic, cationic dye, 5,5′,6,6′-tetrachloro-1,1′,3,3′ tetraethylbenzimidazoly-carbocyanine iodide (JC-1) per manufacturer’s manual (Invitrogen Life Technology, Carlsbad, CA, USA). The JC-1 can freely permeate cells and undergoes reversible transformation from a monomer into an aggregate form when bound with high mitochondrial membrane potential. The JC-1 aggregate form is distinguished from the monomeric form by taking on bright red fluorescence (excitation 530 nm/emission 600 nm) (non-apoptotic cells) in reaction to 488 nm excitation while the monomer on a background of green fluorescence (excitation 490 nm, emission 530 nm) (apoptotic cells). The treated cells were stained with JC-1 (10 μg/mL) stock solution for 20 min prior to be harvested for analysis. After an additional 20 min of incubation, cells were harvested by centrifugation at 1000 rpm for 5 min and washed twice by triple volumes of PBS, and re-suspended in PBS before they were immediately analyzed by flow cytometry (BD FACSCanto II) using appropriate gate settings in FL1 (green) and FL2 (red) channels. For each treatment condition, at least 10,000 cells were statistically analyzed. All the procedures were light proof prior to the cytometry analysis [36].

### 3.11. Immnoblotting Analysis

Whole cell extract were prepared by homogenizing the cells in a lysis buffer (50 mM Tris-HCl, pH 7.5, 5 mM EDTA, 150 mM NaCl, 0.5% NP-40) for 60 min on ice. The lysates were then centrifuged at 10,000× *g* for 10 min at 4 °C, and the supernatants were collected as whole-cell extracts. The soluble protein concentration was measured with Bio-Rad Protein Assay (Bio-Rad Laboratories, Richmond, CA, USA) using bovine serum albumin (BSA) as a standard. The cell extracts (50 μg) were separated by 10% sodium dodecyl sulfate (SDS)-polyacrylamide gel (SDS-PAGE) and transferred to a PVDF membrane (Millipore, Billerica, MA, USA). The membrane was blocked in 4% fat free dry milk in PBS containing 0.2% Tween-20 and probed using antibody against Bcl-2-associated X protein (Bax), cleaved capase-3, NFκB (p65), Myeloid cell leukemia-1 (Mcl-1), β-catenin or cyclin D1 and β-actin followed by appropriate peroxidase labeled secondary antibodies. The blots were then developed using the enhanced chemiluminescence (ECL) reagent (Amersham Biosciences, Piscataway, NJ, USA). All above antibodies were from Protein Tech Group (Chicago, IL, USA), except the antibodies against β-catenin, cyclin D1 and cleaved capase-3, which were products of Millipore (Billerica, MA, USA) and Cell Signaling Technology (Beverly, MA, USA).

### 3.12. Caspase-3 Activity Assay

The enzymatic activity of Caspase-3 was determined using caspase-3 activity kit per manufacturer’s manual (Beyotime Institute of Biotechnology, Haimen, China). The caspase-3 activity was ascertained by measuring the cleavage of colorless substrate specific for caspase-3 (Ac-DEVD-pNA) releasing the chromophore, *p*-nitroaniline (pNA). Assays were performed on 96-well plates by incubating 10 μL protein of cell lysate per sample in 80 μL reaction buffer (1% NP-40, 20 mM Tris-HCl (pH 7.5), 137 mM Nad and 10% glycerol) containing 10 μL caspase-3 substrate (Ac-DEVD-pNA, 2 mM) at 37 °C for 4 h. The samples were then used for measurement of absorbance at 405 nm on an ELISA reader.

### 3.13. Statistical Analysis

All data collected in this study was obtained from at least three independent experiments for each condition. SPSS18.0 analysis software was used for the statistic analysis. Statistical evaluation of the data was performed by one-way ANOVA and t-test for comparison of differences between the two groups. A value *p* < 0.05 set to represent a statistical difference and a value *p* < 0.01 set to represent a statistically significant difference. Data was presented as the mean ± standard deviations (SD).

## 4. Conclusions

In summary, the results reported in this study demonstrate that activation of Wnt/β-catenin signaling is able to promote apoptosis in alveolar macrophage RAW264.7 cells in response to mycobacteria BCG infection. Furthermore, the activated Wnt signal has an ability to promote macrophage apoptosis. In this regard, a caspase-dependent apoptosis pathway is involved in this process, in which anti-apoptosis factor Mcl-1 is down-regulated, and cleaved caspase 3 and Bax are up-regulated. Of note, other apoptosis pathways may also be involved in the Wnt-promoted cell apoptosis, which need to be defined in future study. These observations have implications for the development of improved strategies to target Mtb infection.
